# Selenite-induced ROS/AMPK/FoxO3a/GABARAPL-1 signaling pathway modulates autophagy that antagonize apoptosis in colorectal cancer cells

**DOI:** 10.1007/s12672-021-00427-4

**Published:** 2021-09-24

**Authors:** Hailing Yu, Yin Huang, Yanming Ge, Xiaopeng Hong, Xi Lin, Kexin Tang, Qiang Wang, Yang Yang, Weiming Sun, Yongquan Huang, Hui Luo

**Affiliations:** 1 Guangdong Provincial Key Laboratory of Biomedical Imaging and Guangdong Provincial Engineering Research Center of Molecular Imaging, The Fifth Affiliated Hospital, Sun Yat-Sen University, No.52 of Meihua Dong Road, Xiangzhou District, Zhuhai, Guangdong Province China; 2 The Green Aerotechnics Research Institute of Chongqing Jiaotong University, Chongqing, China; 3 Department of Cardiology, The Fifth Affiliated Hospital, Sun Yat-Sen University, Zhuhai, Guangdong Province China; 4 Department of Pharmacy, The Fifth Affiliated Hospital, Sun Yat-Sen University, Zhuhai, Guangdong Province China; 5 Department of Hepatobiliary Surgery, The Fifth Affiliated Hospital, Sun Yat-Sen University, Zhuhai, Guangdong Province China; 6 Institute of Basic Medical Sciences, Peking Union Medical College, Beijing, China; 7 Department of Ultrasound, The Fifth Affiliated Hospital, Sun Yat-Sen University, No.52 of Meihua Dong Road, Xiangzhou District, Zhuhai, Guangdong Province China

**Keywords:** AMPK, Apoptosis, Autophagy, FoxO3a, Selenite

## Abstract

Previous studies have shown that selenium possessed chemotherapeutic effect against multiple malignant cancers, inducing diverse stress responses including apoptosis and autophagy. Selenite was previously shown to induce apoptosis and autophagy in colorectal cancer cells. However, the relationship between selenite-induced apoptosis and autophagy was not fully understood. Our results revealed a pro-survival role of selenite-induced autophagy against apoptosis in colorectal cancer cells. Real-time PCR array of autophagy-related genes showed that GABARAPL-1 was significantly upregulated in colorectal cancer cells, which was confirmed by western blot and immunofluorescence results. Knockdown of GABARAPL-1 significantly inhibited selenite-induced autophagy and enhanced apoptosis. Furthermore, we found that selenite-induced upregulation of GABARAPL-1 was caused by upregulated p-AMPK and FoxO3a level. Their interaction was correlated with involved in regulation of GABARAPL-1. We observed that activation and inhibition of AMPK influenced both autophagy and apoptosis level via FoxO3a/ GABARAPL-1 signaling, implying the pro-survival role of autophagy against apoptosis. Importantly, we corroborated these findings in a colorectal cancer xenograft animal model with immunohistochemistry and western blot results. Collectively, these results show that sodium selenite could induce ROS/AMPK/FoxO3a/GABARAPL-1-mediated autophagy and downregulate apoptosis in both colorectal cancer cells and colon xenograft model. These findings help to explore sodium selenite as a potential anti-cancer drug in clinical practices.

**Supplementary Information:**

The online version contains supplementary material available at 10.1007/s12672-021-00427-4.

## Introduction

Colorectal cancer, one of the leading causes of death worldwide, is increasing rapidly with the recurrence and metastases of late-stage patients remaining a major problem. Exploring novel strategies in treating colorectal cancer is of great significance. Selenium, which widely exists in daily food and water in the form of selenoproteins, is an essential trace element for human health. Studies show that selenium possesses chemopreventive and chemotherapeutic effects against prostate, lung, colorectal and bladder cancers [1]. Sodium selenite, an important inorganic form of selenium, has been shown to induce both apoptosis and autophagy of cancer cells, however, their crosstalk and detailed molecular mechanism is unclear [2]. Elucidating the contribution of selenite-induced autophagy toward apoptosis and the precise interplay between autophagy and apoptosis would facilitate clinic application of selenite and identifying targets for combinational therapy.

Autophagy, characterized by enclosure of organelles and cytoplasm component inside double membrane vesicles that subsequent to degradation, is one of the early adaptive response towards stress [3, 4]. The process is directed by multiple distinct autophagy-related (Atg) proteins that participate in the formation of a specialized double-membraned organelle, which is called autophagosome, enabling vesicle nucleation, elongation, cargo recruitment and closure [5, 6].

Whether autophagy promotes or inhibits cell death and its function in tumorigenesis remain highly controversial. At physiological level, autophagy can provide energy under starvation, and clear up protein aggregates as well as damaged organelles, functioning as a cytoprotective mechanism that favors stress adaption thus avoid cell death [7, 8]. However, autophagic cell death can also occur with unique morphological changes and biochemical characters. In addition, there is also report that autophagy can selectively remove survival factors thus favors cell death [9]. Multiple research suggests interconnection between autophagy and apoptosis exist, however, it’s unclear whether the autophagy antagonizes apoptosis or promotes apoptosis [10]. Some report calpain and caspases can convert autophagy to apoptosis by cleaving autophagy-related protein like Beclin1 and Ambra1 [11, 12], while others found autophagy to be beneficial and pro-survival to cells by eliminating damaged organelles and proapoptotic substances [13, 14].

Our previous research showed that selenite triggered opposite patterns of autophagy in different leukemia cell lines during apoptosis [15], while selenite induced coherent autophagy that antagonized apoptosis in vivo and in vitro [16]. To investigate the key proteins in selenite-induced protective autophagy, reverse transcription (RT)-PCR autophagy microarray was performed to screen from 84 key genes including autophagy machinery components and regulators to identify potential targets, and the result showed that GABARAPL-1 played a vital role in selenite-induced autophagy. GABARAPL-1 is one member of the GABARAPL subfamily that involved in mediating the function of membrane receptors such as the GABA receptor and intracellular protein transport [17]. GABARAPL-1, which responsible for the maturation of autophagosomes, interacts with autophagy receptors such as p62 and NBR1, allowing degradation of protein aggregates that binds to autophagy receptors [18]. Interestingly, our research identified the key role of GABARAPL-1 in selenite-induced autophagy as well as apoptosis.

In this study, we investigated in detail the crosstalk between selenite-induced autophagy and apoptosis in colorectal cancer cells and elucidated the role of ROS/AMPK/FoxO3a/GABARAPL-1 signaling pathway in regulating protective autophagy over apoptosis in both colorectal cancer cell lines and xenograft tumor model.

## Results

### Selenite treatment induced GABARAPL-1 expression, which is involved in autophagy

**Fig. 1:**
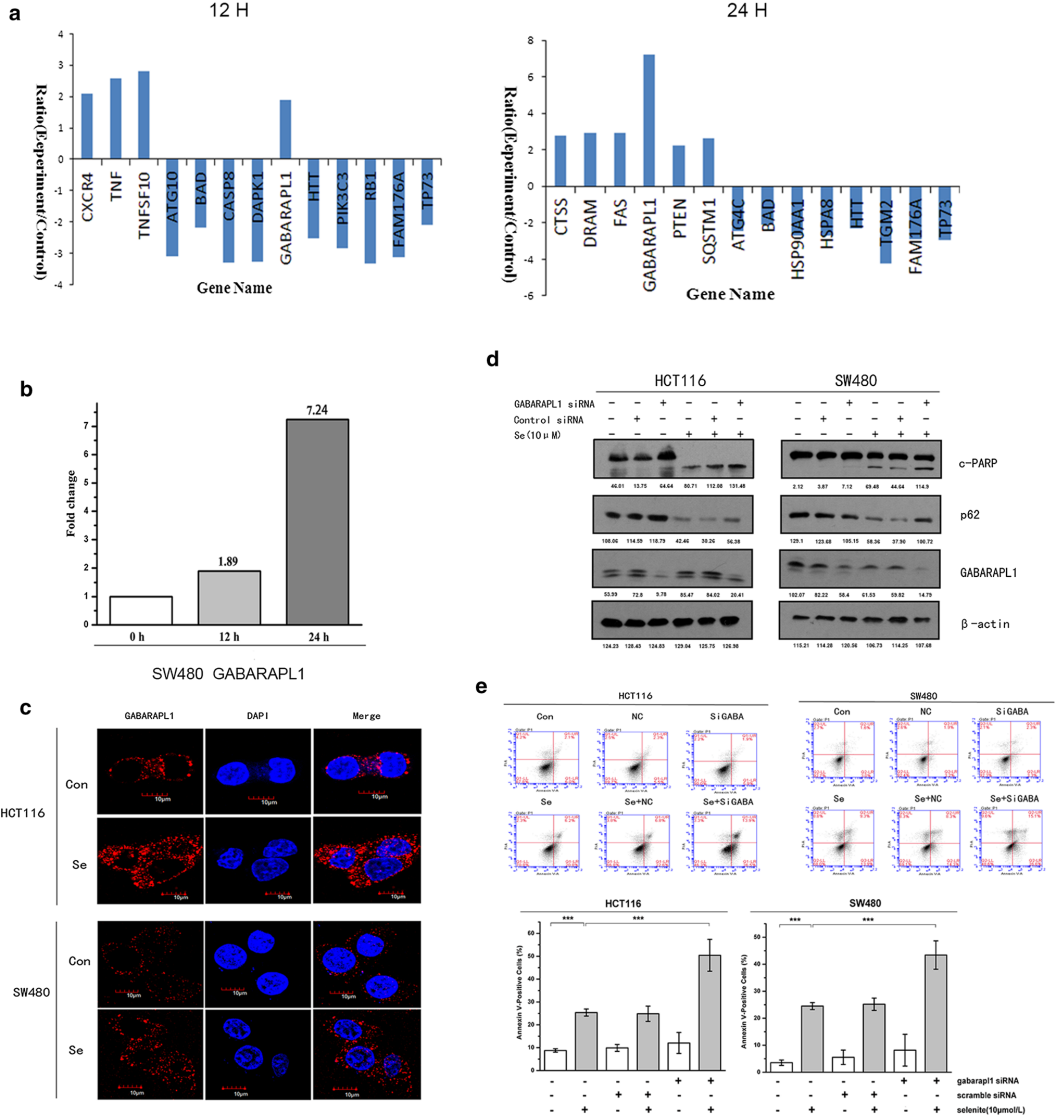
GABARAPL-1 was upregulated by selenite treatment and was associated with autophagy and apoptosis of CRC cells. **a** Expression of genes markedly changed after selenite treatment in SW480 cells for 12 and 24 h were shown in the graph. **b** mRNA expression level of GABARAPL-1 after selenite treatment. **c** Confocal analysis of GABARAPL-1 protein level in selenite-treated SW480 and HCT116 CRC cells. The cells were incubated with primary antibody against GABARAPL-1 and Cy3-conjugated secondary antibody (red). The nuclei were stained with DAPI. Scale bar, 10 µm. **d**, **e** HCT116 and SW480 cells were treated with siRNA targeting GABARAPL-1 and subjected to western blot analysis or FACS analysis. The statistical graphs are presented as the mean ± S.D. of the three independent experiments, *p < 0.05

Our previous results show that supranutritional doses of sodium selenite induce both apoptosis and autophagy in colorectal cancer cells. Autophagy and apoptosis are two conserved physiological stress responses elicited by sodium selenite. To explore the detailed mechanism of selenite-induced crosstalk between autophagy and apoptosis, Real-time PCR array was performed to screen candidate genes involved in this process. Specifically, mRNA level in SW480 cells of 84 autophagy-related genes was analyzed before and after selenite treatment for 12 and 24 h, respectively. Among the 27 genes that exhibit more than two fold change after selenite treatment (Fig. 1a and Supplementary Fig. 1), GABARAPL-1 expression significantly increased over time 24 h. (Fig. 1b). Furthermore, immunostaining shows that GABARAPL-1 protein level also increased upon selenite treatment for 24 h (Fig. 1c). Western blot assays confirmed selenite treatment induced elevated GABARAPL-1 protein level and downregulation of p62 shown in Fig. 1d, indicating enhanced autophagy. To investigate the role of GABARAPL-1 in selenite-induced crosstalk of autophagy and apoptosis, we silenced GABARAPL-1 in HCT116 and SW480 cells. As shown in Fig. 1d, silencing GABARAPL-1 decreased selenite-induced autophagy as indicated by elevated p62 accumulation. Conversely, enhanced PARP cleavage was detected upon GABARAPL-1 inhibition, indicating increased caspases activity during apoptosis. In addition, FACS experiment confirms that inhibiting GABARAPL-1 exacerbated selenite-induced apoptosis, increasing from 27.0 to 50.4% and from 23.2% to 41.7% in HCT116 and SW480 respectively. (Fig. 1e). Collectively, the results show that sodium selenite-induced upregulation of GABARAPL-1 promoted autophagy, which exerted a protective role against apoptosis.

### Selenite treatment activated AMPK/FoxO3a/ GABARAPL-1 signaling

**Fig. 2:**
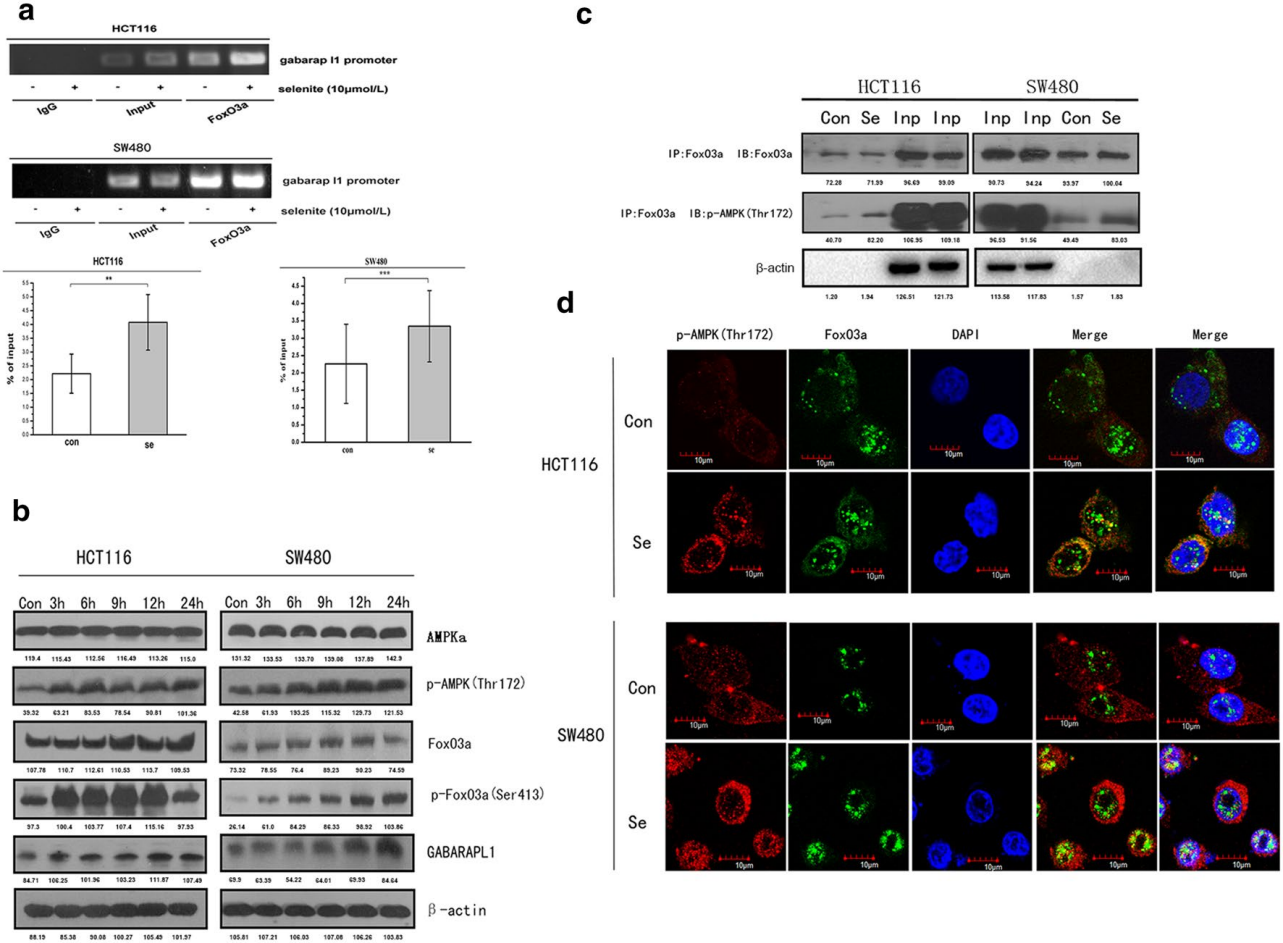
AMPK activated FoxO3a responsible for GABARAPL-1 transcription. **a** Selenite treatment enhanced FoxO3a binding to gabarapl-1 promoter. The statistical graphs are presented as the mean ± S.D. of the three independent experiments, *p < 0.05. **b** Western blot analysis of AMPK and FoxO3a protein levels and phosphorylation levels during a 0–24 h time course in HCT116 and SW480 cells treated with 10 µM selenite. **c** Enhanced p-AMPK and FoxO3a interaction after selenite treatment. Co-immunoprecipitation experiment was conducted with FoxO3a antibody and subjected to western blot experiments incubated with p-AMPK or FoxO3a antibody. β-actin was used as a loading control. **d** Confocal analysis of p-AMPK and FoxO3a interaction in selenite-treated HCT116 and SW480 cells. Cells were incubated with primary antibody against p-AMPK and FoxO3a then stained with Cy3 or FITC-conjugated secondary antibody. The nuclei were shown as blue signals. Scale bar, 10 µm

AMP-activated protein kinase (AMPK) is an adenine nucleotide sensor that plays a key role in the regulation of energy homeostasis as well as energy-producing events including autophagy [19, 20].While selenite treatment induced autophagy to provide energy for cell survival, we investigated the potential role of AMPK in selenite-induced autophagy. To begin with, we examined the expression of p-AMPK (Thr172), an active form of AMPK and its downstream molecule p-FoxO3a (Ser413) [21]. As shown in Fig. 2b, we found that sodium selenite upregulated p-AMPK and p-FoxO3a expression in a time-dependent manner. Furthermore, we analyzed AMPK and FoxO3a interaction by co-immunoprecipitation and immunofluorescence experiments. Co-immunoprecipitation experiments show that FoxO3a binds with p-AMPK in CRC cells. p-AMPK bands were observed in samples immunoprecipitated with anti-FoxO3a antibodies both control and selenite-treated group. Moreover, after being normalized to β-actin, the interaction of FoxO3a with p-AMPK increased after selenite treatment as shown in the second and the eighth band of Fig. 2c. We also found increased colocalization of p-AMPK with FoxO3a in selenite-treated samples from immunofluorescence experiments in both HCT116 and SW480 cells (Fig. 2d). Since FoxO3a is known to enhance GABARAPL-1 transcription by binding to its promoter [22], we investigated the role of FoxO3a on GABARAPL-1 expression upon selenite treatment. Chromatin immunoprecipitation (ChIP) experiment was conducted to analyze the binding of FoxO3a to GABARAPL-1 promoter. After sodium selenite treatment for 24 h, the binding of the transcription factor FoxO3a to GABARAPL-1 promoter increased significantly compared with control (Fig. 2a and Supplementary Fig. 2). Altogether, these results show that sodium selenite treatment activated AMPK/FoxO3a signaling that enhanced the transcription of GABARAPL-1.

### AMPK/FoxO3a signaling modulates the crosstalk between autophagy and apoptosis in colorectal cancer cells

**Fig. 3:**
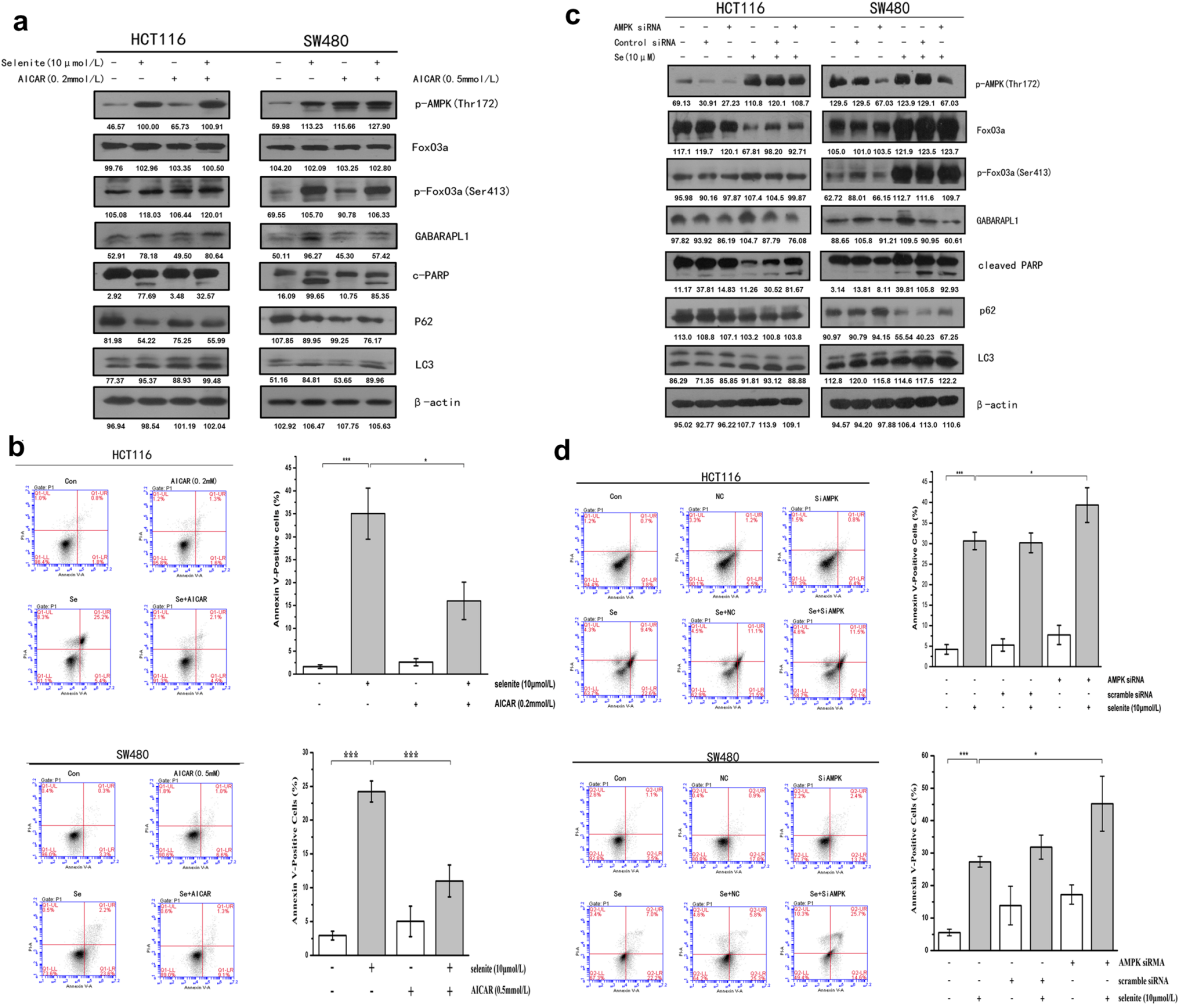
Selenite-induced AMPK-dependent autophagy protected cells against apoptosis. **a** and **b** Activating AMPK enhanced selenite-induced autophagy and attenuated apoptosis. 0.2 mM AMPK activator AICAR was added to HCT116 cells and 0.5 mM AICAR was added to SW480 cells 2 h prior to 10 µM selenite treatment. Samples were collected and subjected to western blot analysis incubated with indicated antibodies or FACS analysis. **c** and **d** Silencing AMPK reduced selenite-induced autophagy and promoted apoptosis. HCT116 and SW480 cells were transfected with siRNA targeting AMPK before selenite treatment and subjected to western blot or FACS analysis. The statistical graphs are presented as the mean ± S.D. of the three independent experiments, *p < 0.05

Based on above findings, we tentatively investigate the role of AMPK/FoxO3a signaling in selenite-induced autophagy and apoptosis. We modulated the activity of AMPK by its activator AICAR or AMPK siRNA. Cells were treated with AMPK activator AICAR for 2 h before selenite treatment for 24 h. Total cell lysates were collected and subjected to western blot assays. Selenite treatment increased phosphorylation and activation of p-AMPK which is further enhanced by AICAR pretreatment. Moreover, FoxO3a phosphorylation at Ser 413 and GABARAPL-1 expression were also enhanced when cells were treated with selenite combined with AICAR. While AICAR pretreatment enhanced selenite-induced autophagy as indicated by autophagy marker LC3 and P62, AICAR attenuated selenite-induced apoptosis in both cell lines as indicated by less cleaved PARAP (Fig. 3a). FACS experiments also confirmed that AMPK activator AICAR pretreatment significantly attenuated selenite-induced apoptosis, decreasing the apoptotic rate from 30.6 to 6.6% and from 25.8 to 10.4% in HCT116 and SW480 cells respectively. (Fig. 3b). By contrast, silencing AMPK by siRNA decreased the expression of p-FoxO3a and GABARAPL-1 and inhibited subsequent autophagy as shown by increased p62 and decreased LC3 conversion, while enhanced selenite-induced apoptosis as indicated by enhanced cleaved PRAP observed from western blot assays (Fig. 3c). FACS experiments consistently show that AMPK knockdown increased apoptotic rate from 32 to 36.6%, and 29.2 to 40.3% in HCT116 and SW480 cells, respectively. (Fig. 3d). These results collectively demonstrate that selenite-induced AMPK/FoxO3a/GABARAPL-1-dependent autophagy protected colorectal cancer cells against apoptosis.

### AMPK/FoxO3a/GABARAPL-1 signaling was modulated by sodium selenite in colorectal xenograft model

**Fig. 4:**
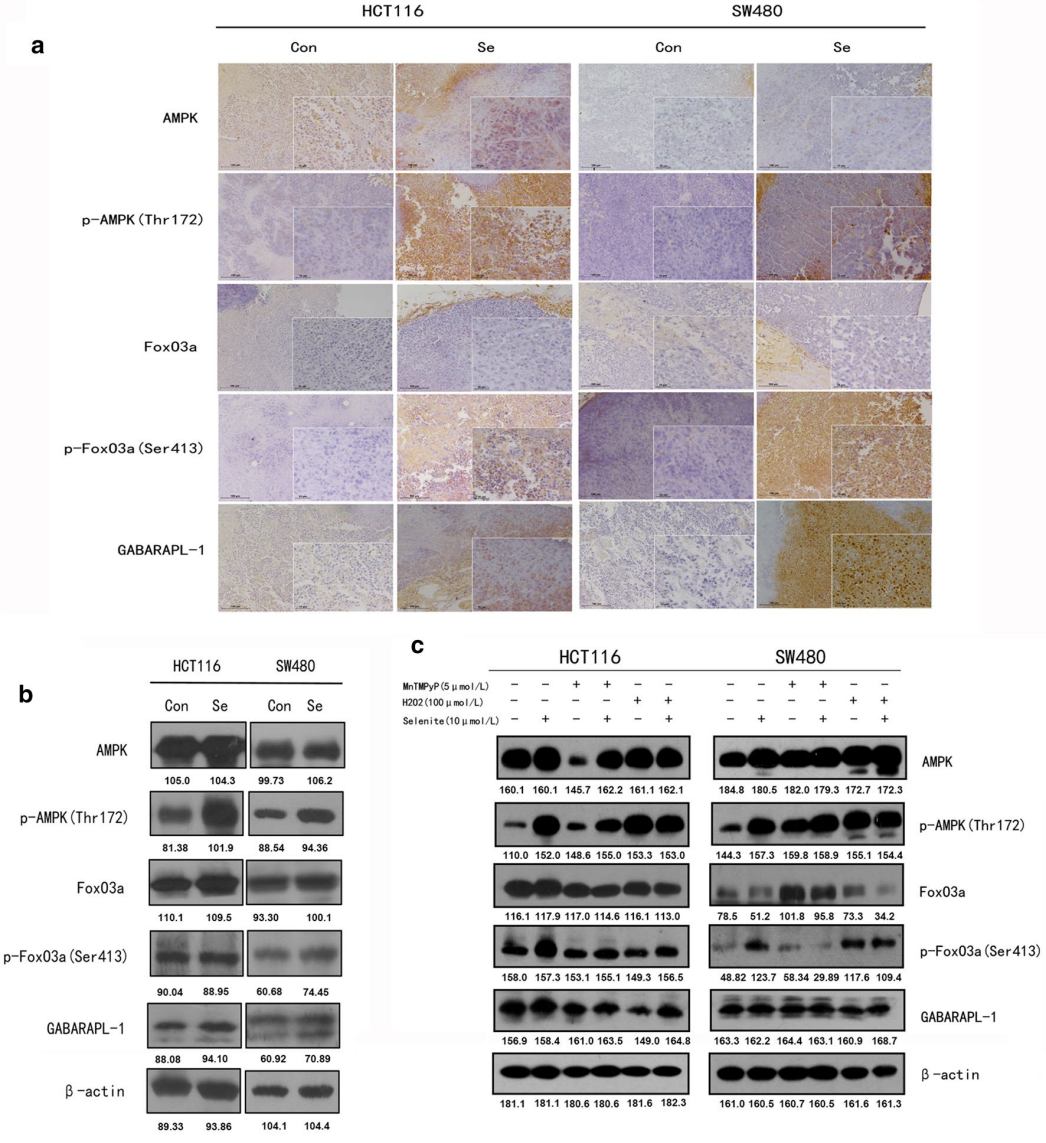
Selenite activated AMPK/FoxO3a/GABARAPL-1 signaling in colorectal cancer xenograft model. **a** Immunohistochemistry results of HCT116 and SW480 xenograft model with antibodies for AMPK, p-AMPK, FoxO3a, p- FoxO3a and GABARAPL-1. Scale bar, 100 µm and 25 µm for the larger picture and boxed area, respectively. **b** Western blot analysis of the tissue sample. **c** Influence of ROS depletion on AMPK/FoxO3a/GABARAPL-1 signaling. Cells were pretreated with 5 µM MnTMPyP 2 h before selenite treatment

Based our results on cell lines, we further aim to investigate role of selenite-induced AMPK/FoxO3a/GABARAPL-1 signaling on in vivo model. We subcutaneously inoculated HCT116 and SW480 cells on nude mice. We examined the expression of p-AMPK, p-FoxO3a and GABARAPL-1 in tissues from both HCT116 and SW480 colorectal xenograft model. Immunohistochemistry results showed that p-AMPK, p-FoxO3a, GABARAPL-1 level increased upon selenite treatment, while AMPK and FoxO3a expression remained almost constant after selenite treatment (Fig. 4a). Consistently, western blot results also confirmed activated AMPK/FoxO3a/GABARAPL-1 signaling pathway upon selenite treatment in HCT116 and SW480 colorectal xenograft model (Fig. 4b). These results pointed out that AMPK/FoxO3a/GABARAPL-1 signaling was involved in colorectal xenograft model, validating our in vitro results.

### Reactive oxygen species (ROS) was involved in selenite-modulated AMPK/FoxO3a/GABARAPL-1 signaling

Previous results have shown that ROS was an important upstream regulator of selenite-induced cell death [2]. To address this concern, we aim to investigate the role of ROS in selenite-regulated AMPK/FoxO3a/GABARAPL-1 signaling, we applied MnTMPyP, which is a ROS scavenger and H_2_O_2_ which is a ROS inducer to modulate ROS level in HCC cells. As shown in Fig. 4c, MnTMPyP pretreatment attenuated selenite-induced upregulation of p-FoxO3a, p-AMPK and GABARAPL-1. As a positive control, H_2_O_2_ alone increased p-AMPK, FoxO3a and GABARAPL-1. Collectively, these results show that ROS was an upstream regulator of selenite-modulated AMPK/FoxO3a/ GABARAPL-1 signaling and the crosstalk between apoptosis and autophagy.

## Discussion

Our previous work found that selenite can exert an effect on both autophagy and apoptosis in leukemia cells [23, 24]. However, the crosstalk between autophagy and apoptosis remains unknown. In this research, we modified activity or expression level of autophagy-related proteins and observed changes in apoptotic rate in selenite-treated colorectal cancer cells. We delineated the correlation of autophagy and apoptosis induced by selenite in CRC cells under the regulation of AMPK/FoxO3a signaling pathway in this study.

Atg8 that responsible for the formation of autophagosomes is comprised of two subfamilies: GABARAPL and LC3. The covalent ligation of phosphatidylethanolamine (PE) to Atg8 regulates a variety of process during autophagy including recruiting cargos and regulatory proteins to its marked membranes as well as autophagosome biogenesis [6]. Previous results have shown that GABARAPL-1 knockdown leads to decreased autophagy flux and lysosome number as well as increased cell growth [25], while GABARAPL-1 overexpression inhibits cancer cell proliferation and tumor formation in nude mice via Wnt/β-catenin pathway [26]. However, it is unclear how GABARAPL-1 regulates both autophagy and tumor growth. Our PCR Array result found that GABARAPL-1 gradually increased upon selenite treatment together with upregulated autophagy and apoptosis level. Interrupting GABARAPL-1 activity can affect both autophagy and apoptosis in colorectal cancer cells, suggesting crosstalk between the two processes. AMPK is a well-established energy sensor that promotes cell proliferation and growth via providing energy and inhibiting anabolic processes [19]. AMPK has been reported to regulate FoxO3a indirectly and directly under different stimulations [27, 28]. Here, our results found that AMPK can directly bind to FoxO3a via co-immunoprecipitation and immunofluorescence experiments in colorectal cancer cells after selenite treatment. Our research adds new insights into the mechanism of how AMPK activated FoxO3a.

Research by Kralova et al*.* also reveals the crosstalk between autophagy and apoptosis in selenite-treated HCT116 cells [29]. Presence of autophagy was reported in colorectal cancer cells upon selenite treatment as characterized by vacuolization in cell cytoplasm before apoptosis occurred. In accord with our conclusion [16], their results show that suppression of selenite induced autophagy with 3-MA, which blocks initiation of autophagy, induced significant increase of toxicity and apoptosis. Meanwhile, our research found that silencing GABARAPL-1 which participates in autophagosome formation [17] or inhibiting AMPK which is vital in autophagy induction[20] can increase selenite-induced apoptosis. However, Kralova et al*.* wasn’t able to detect influence of selenite induced apoptosis in HCT116 cells using rapamycin, a selective mTOR inhibitor [29]. Interestingly, CB1 modulated autophagic flux was reported in certain physiological situations independent of mTOR [30]. Additionally, mTOR-independent non-canonical autophagy was reported to provide stress resistance to neuroblastoma and breast cancer cells [31], suggesting selenite may induce protective autophagy in HCT116 cells via AMPK/GABARAPL-1 related pathways without affecting mTOR. To conclude, our results show selenite induced autophagy though distinct pathways to protect cells against subsequent apoptosis in colorectal cancer cells, which is consistent with previous research results.

The Forkhead box O (FoxO) family is known as important transcriptional regulator with FoxO1, FoxO3a, FoxO4 and FoxO6 identified as family members in humans. FoxO3a has been extensively studied for its vital role in apoptosis, stress management and metabolism [32]. Our previous result also showed that Akt/FoxO3a was activated upon selenite treatment [2], which is consistent with reports that FoxO3a triggers both apoptosis and autophagy in different scenarios [33]. In this study, we reveal a novel role of FoxO3a regulated by AMPK in promoting autophagy. Of note, the PI3K-Akt pathway is reported to activate autophagy by two mechanisms: mTOR- dependent pathway and FoxO3 mediated mTOR-independent pathway in which FoxO3 stimulate autophagy via enhancing transcription of LC3 and GABARAPL-1[22]. This could also explain why selenite-induced autophagy in HCT116 cells is independent of mTOR in previous study [29].

The mechanism of autophagy and apoptosis crosstalk is not fully understood. Multiple researches report that calpain and caspases cleave autophagy-related proteins thus switch autophagy to apoptosis, however, only the truncated form of Atg5 is found to translocate from cytosol to mitochondria and provoke subsequent mitochondria apoptosis pathways [34]. Other studies found that proteolysis of autophagy-related protein Beclin-1, Ambra 1 and Atg4D inhibit autophagic pro-survival response [9, 11, 35]. In our research, by interrupting autophagy-related protein AMPK and GABARAPL-1, we observed corresponding influence in apoptosis, providing additional evidence for pro-survival role of autophagy. Importantly, our research shows a protective role of selenite-induced autophagy, suggesting that proteins participate in both autophagy and apoptosis should be avoid in designing molecule based drugs in order to achieve a maximum effect.

In conclusion, our study identifies a signaling pathway that responds to selenite-induced protective autophagy to combat against apoptosis. Selenite induced ROS production promoted phosphorylation of AMPK, which binds and activates FoxO3a. The phosphorylated FoxO3a binds to GABARAPL-1 promoter thus upregulate GABARAPL-1 level at transcription level, facilitating autophagy that serves as a survival signal against apoptosis.

## Conclusions

We discovered that sodium selenite treatment induced the crosstalk between autophagy and apoptosis through regulating AMPK/FoxO3a/GABARAPL1 signaling pathway in colorectal cancer cells. ROS was an inducer of downstream signaling hub elicited by sodium selenite. Supranutritional doses of sodium selenite might bear potential in clinical application against colorectal cancer through modulating the crosstalk between apoptosis and autophagy elicited by selenite.

## Materials and methods

### Cell lines and culture

HCT116 and SW480 CRC cells were cultured in DMEM supplemented with 10% fetal bovine serum and antibiotics (100 units/ml penicillin and 100 mg/ml streptomycin) at 37 °C in a 5% CO_2_ humidified environment.

### Reagents and antibodies

Sodium selenite, actin and GABARAPL1 antibodies were purchased from Sigma-Aldrich (St. Louis, MO, US, HPA051386 for immunohistochemistry analysis and SAB2013117 for western blot). Antibodies for P62, PARP, LC3, FoxO3a, p-FoxO3a, AMPK, p-AMPK and AMPK activator AICAR were purchased from Cell Signaling Technology (Danvers, MA, USA). Antibodies against AMPK and FoxO3a for immunohistochemistry were purchased from Bioworld Technology (St. Louis Park, MN, USA).

### Immunoprecipitation

The cells were harvested and washed with PBS and then lysed in RIPA buffer with protease inhibitors on ice for 1 h. After centrifugation at 17 000 × *g* for 15 min, the supernatants were collected and adjusted to the same concentration. 2% input sample was set aside. Either primary antibody (2 μl) or normal immunoglobulin antibody as control were added to 100 μl cell lysates and rotated overnight at 4 °C. Then, 30 μl protein A + G agarose beads (Santa Cruz Biotechnology, Inc.) were added to the mixture and rotated at 4 °C for 3 h. The target protein and its complex were collected at 4000 rpm for 5 min at 4 °C and washed four times with RIPA buffer. RIPA buffer with protein loading dye was mixed to the pellet, which was subjected to western blot analysis together with the 2% input sample.

### Western blot

Whole-cell pellets were lysed in RIPA buffer with protease inhibitors and sonicated to allow complete lysis. Bradford assay was used to determine the protein concentration. Protein samples were loaded and subjected to SDS–polyacrylamide gel electrophoresis and transferred to nitrocellulose membranes, which were blocked in 5% skim milk in Tris-buffered saline–Tween-20 and incubated overnight with primary antibodies at 4 °C. Secondary antibodies were incubated with a 1: 5000 dilutions. SuperSignal West Pico Chemiluminescent Substrate (Thermo Fisher, Waltham, MA, USA) was used to detect the signal. Auto contrast was applied for scanned membrane. Band intensity was quantified using Image J software.

### Immunofluorescence

Cells were seeded onto coverslips and treated with selenite for 24 h. After fixing with 5% paraformaldehyde for 10 min, the cell membranes were permeabilized with 0.2% Triton for 30 min. After blocked with 1% BSA for 1 h to avoid nonspecific binding, cells were stained with primary antibodies at 4 °C overnight and with Cy3- or FITC-conjugated secondary antibodies for 1 h at room temperature. The slides were preserved using Mounting Medium with DAPI (Vectashield, Burlingame, CA, USA). Images were acquired using an Olympus laser scanning confocal FV1000 microscope (Olympus, Tokyo, Japan) and analyzed with Olympus Fluoview software.

### ChIP assay

Chromatin extracts were immunoprecipitated using an antibody against FoxO3a. The target chromatin was extracted and immunoprecipitated using the SimpleChIP Enzymatic Chromatin IP Kit (Cell Signaling Technology, Catalogue no. 9002 s) according to the manufacturer’s instructions. Primers used to amplify the immunoprecipitated chromatin (Forward 5’- GAAAACCAAGAAGTGGGCTATG-3’; Reverse 5’- TTCAAGGATGCTTTGTGCTG-3’) were synthesized by Sangon Biotech (Shanghai, China).

### siRNA transfection

AMPK siRNA (5’-GGUUGGCAAACAUGAAUUGtt-3’) and non-targeting control siRNA were acquired from GenePharma (Shanghai, China). GABARAPL1 siRNA was purchase from Santa Cruz Biotechnology, Inc. (Dallas, TX, USA). Transfections were performed using Lipofectamine 2000 (Invitrogen) according to the recommended procedure. The cells were plated onto six-well dishes at a density of 4 × 10^5^ cells per well the day before transfection. 3 μl Lipofectamine 2000 was combined with 4000 pmol (20 μl of a 20 μM stock) siRNA in a volume of 500 μl Opti-MEM Media (Gibco) and incubated for 20 min before being added to each well.

### RT^2^ Profiler PCR Array

SW480 total RNA was extracted with TRIzol reagent (Invitrogen) according to the manufacture’s instruction. RNA purity was quantified and estimated using NanoDrop spectrophotometer and sent to QIAGEN (Hilden, Germany) for PCR Array analysis with reverse transcription reaction followed by high-performance q-PCR analysis of 84 autophagy-related genes. The data was analyzed using web-based analysis tool.

### Flow cytometry analysis

The assay was performed using Alexa Fluor 488 Annexin V/Dead Cell Apoptosis Kit (Invitrogen) according to the manufacturer’s instructions. The harvested cells were incubated in binding buffer with Annexin V and propidium iodide for 15 min and then subjected to detection by Accuri C6 flow cytometer (Accuri Cytometers Inc., Ann Arbor, MI, USA).

### Immunohistochemistry

Animal model for SW480 and HCT116 cells was established previously [36, 37]. Tissues were embedded in paraffin and tissue sections were prepared on slides, dewaxed and rehydrated in xylene and graded alcohols. Antigen retrieval was achieved by heating the slides in a 95 °C water bath with 0.01 mol/l citrate buffer at pH 6.0 for 20 min. Endogenous peroxidase activity was quenched by incubation in 3% hydrogen peroxide solution (Zhongshan Gold Bridge, Beijing, China). The slides were incubated with primary antibodies overnight at 4 °C. Then samples were incubated with a streptavidin–peroxidase complex for 1 h at room temperature. Diaminobenzidine working solution was applied, and the slides were counterstained with haematoxylin.

### Animal experiments

HCT116 and SW480 cells were subcutaneously inoculated in the left shoulder of 6-week-old male Balb/c nude mice. Forty days after inoculation, the tumors were harvested and photographed. Tumor tissue samples were subjected to IHC and western blot assays using the indicated antibodies.

### Statistical analyses

Experiments were repeated at least three times. For the quantitative analyses represented in the histograms, the values are expressed as the mean ± S.D. The significance of differences between mean values was assessed using Student’s *t*-test. All computations were calculated using Microsoft Excel.

## Supplementary Information

**Additional file 1**: **Fig. S1**. Genes that changed at least two fold after selenite-treatment for 12h or 24h were shown in the graph.

**Additional file 2**: **Fig. S2**. Amplication curves of qPCR analysis of gabarapL-1 in ChIP experiments. Left: HCT116; Right: SW480 cells, respectively. GAPDH was used an internal control of templates loading.

## Data Availability

The data analyzed during the current study are available from the corresponding author upon reasonable request.

## Abbreviations

GABARAPL-1: Gamma-Aminobutyric Acid Receptor-Associated Protein-Like 1
AMPK: AMP-activated protein kinase
ROS: Reactive Oxygen Species
CRC: Colorectal cancer
FoxO3a: Forkhead Box O3
FACS: Fluorescence activated cell sorting
