# Supplementary figures and images for: Selenite-induced ROS/AMPK/FoxO3a/GABARAPL-1 signaling pathway modulates autophagy that antagonize apoptosis in colorectal cancer cells
Source: Discov Oncol. 2021 Sep 24;12:35. doi: 10.1007/s12672-021-00427-4 (PMC8777540; doi:10.1007/s12672-021-00427-4)

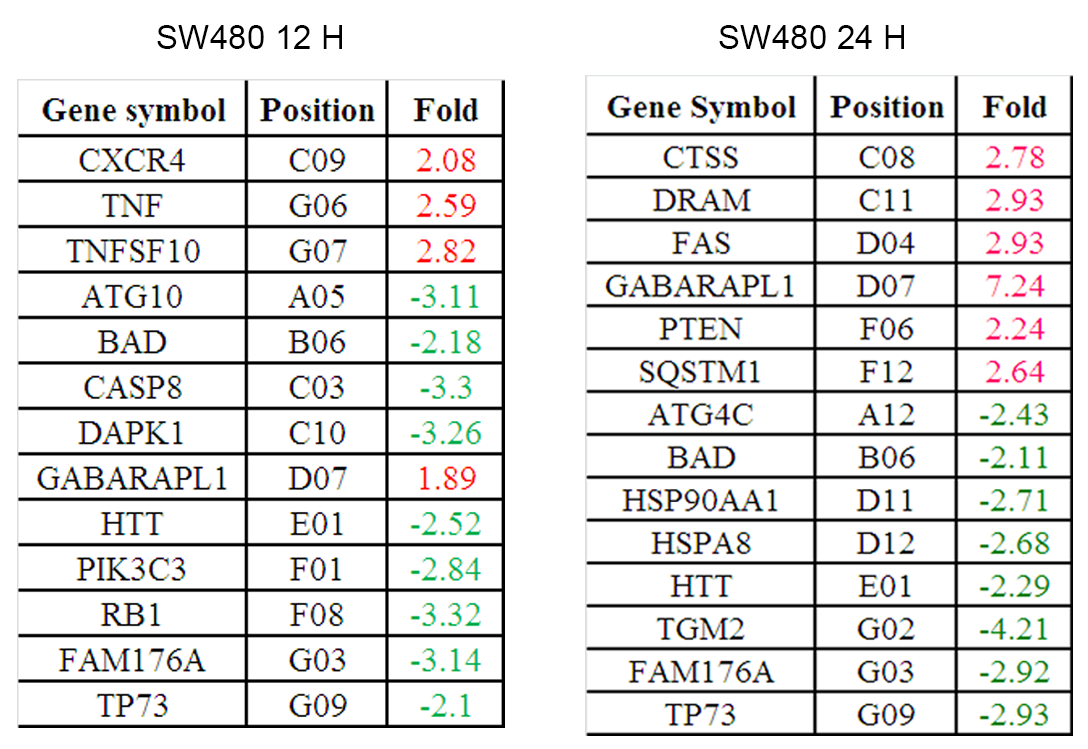

Supplement: Supplementary file 1 — Additional file 1: Fig. S1. Genes that changed at least two fold after selenite-treatment for 12h or 24h were shown in the graph. [file 12672_2021_427_MOESM1_ESM.tif]

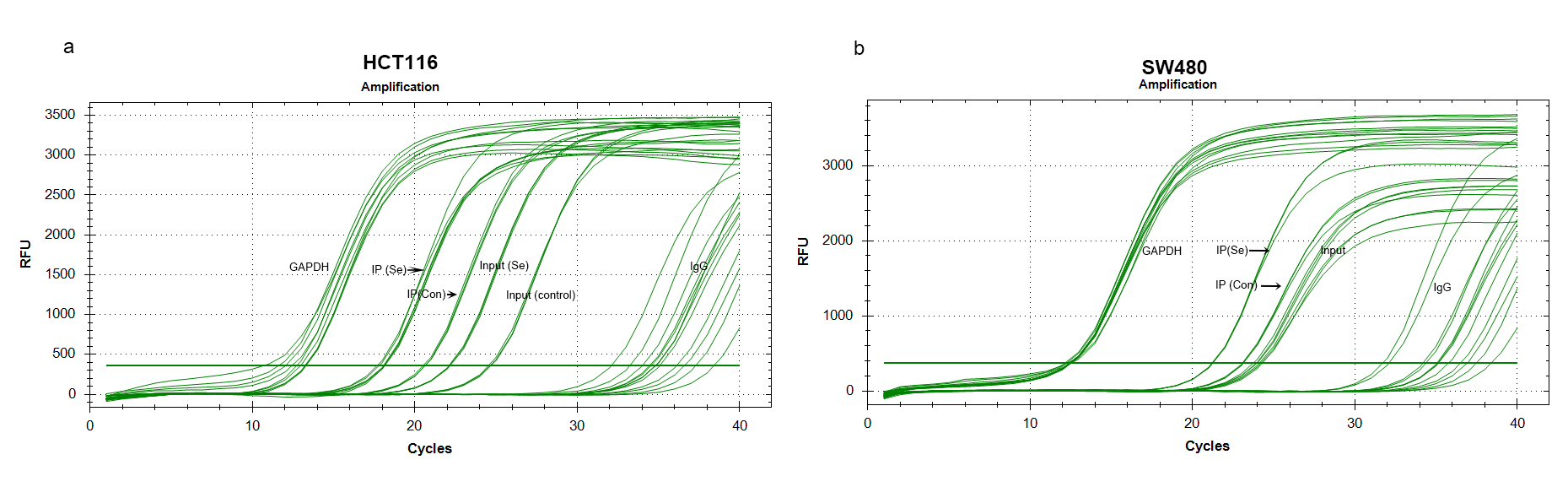

Supplement: Supplementary file 2 — Additional file 2: Fig. S2. Amplication curves of qPCR analysis of gabarapL-1 in ChIP experiments. Left: HCT116; Right: SW480 cells, respectively. GAPDH was used an internal control of templates loading. [file 12672_2021_427_MOESM2_ESM.tif]
